# A subset of bone marrow stromal cells regulate ATP-binding cassette gene expression via insulin-like growth factor-I in a leukemia cell line

**DOI:** 10.3892/ijo.2014.2569

**Published:** 2014-07-29

**Authors:** NADIA BENABOU, PEZHMAN MIRSHAHI, CAMILE BORDU, ANNE-MARIE FAUSSAT, RUOPING TANG, AMU THERWATH, JEANETE SORIA, JEAN-PIERE MARIE, MASSOUD MIRSHAHI

**Affiliations:** 1 UMR, Paris Diderot, Paris 7 University, Lariboisiere Hospital, INSERM U965, Paris, France; 2 Department of Hematology, Saint-Antoine Hospital, Paris, France; 3 Tumor Bank ‘Leukemia’, Saint-Antoine Hospital, Paris, France

**Keywords:** IGF signaling pathway, ATP binding cassette, hospicells, chemoresistance, leukemia

## Abstract

The importance of the insulin-like growth factor, IGF, as a signaling axis in cancer development, progression and metastasis is highlighted by its effects on cancer cells, notably proliferation and acquired resistance. The role of the microenvironment within which cancer cells evolve and which mediates this effect is far from clear. Here, the involvement of IGF-I in inducing multidrug resistance in a myeloid leukemia cell line, grown in the presence of bone marrow-derived stromal cells called ‘Hospicells’ (BMH), is demonstrated. We found that i) drug sensitive as well as resistant leukemia cells express IGF-I and its receptor IGF-IR. However, the resistant cells were found to secrete high levels of IGF-I. ii) Presence of exogenous IGF-I promoted cell proliferation, which decreased when an inhibitor of IGF-IR (picropodophyllin, PPP) was added. iii) BMH and IGF-I are both involved in the regulation of genes of the ATP binding cassette (ABC) related to resistance development (MDR1, MRP1, MRP2, MRP3 and BCRP). iv) The levels of ABC gene expression by leukemia cells were found to increase in the presence of increasing numbers of BMH. However, these levels decreased when IGF-IR was inhibited by addition of PPP. v) Co-culture of the drug-sensitive leukemia cells with BMH induced protection against the action of daunorubicin. This chemoresistance was amplified by the presence of IGF-I whereas it decreased when IGF-IR was inhibited. Our results underline the role of microenvironment in concert with the IGF-1 pathway in conferring drug resistance to leukemia cells.

## Introduction

A major problem in the treatment of leukemia is the appearance of multi-drug resistance (1). Chemoresistance often is at the origin of a major number of relapses which are mediated by the expulsion activity of the ABC pumps.

The microenvironment is intimately related to the development of drug resistance in cancer cells. However, the precise nature of molecular events in action between the stromal and leukemic cells remains still elusive. Chemoresistance of leukemic cells, besides the microenvironment is also controlled by the ATP binding cassette genes (ABC), notably ABCB1 (MDR1) which code for the P-gp protein. In a rather unorthodox series of experiments we identified recently the presence of a distinct population of stromal cells in the tumor microenvironment which we baptized ‘Hospicells’ (BMH). These cells were found to establish very particular relationship with HL60 (acute leukemia cell lines), MDA-MB-231-GFP (breast cancer cell line) and OVCAR-3GFP (ovarian cancer cell line) (2–7). In solid tumors, the very presence of Hospicells were found to confer drug resistance to cancer cells during chemotherapy via oncologic trogocytosis (2,3), as a result of upregulation of ATP binding cassette gene family (4).

Hospicells by their presence promotes tumorigenicity and angiogenesis (5) besides influencing the immune response of tumor microenvironments (6,7). A plethora of cell metabolic functions such as vasculogenic mimicry, cancer cell proliferation, inhibition of cell death and metastasis are known today to be influenced by IGF-1 (8–20).

This is the first report concerning the effect of IGF-I on the regulation of the ABC genes (*MDR1*, *MRP1*, *MRP2*, *MRP3*, *MRP5* and *BCRP*) in leukemic cells when they are put in contact with a subset of bone marrow derived stromal cell named Hospicells.

## Materials and methods

### Cell culture

Four cell lines were used in this study, HL-60, (acute promyelocytic leukemia ATCC, CCL-240), K-562 (chronic myelogenous leukemia ATCC, CCL 243), K562 and HL60 derived from K562/Dox and HL60/Dnr, which were developed as daunorubicin and doxorubicin resistant lines, respectively. These cells are deposited at the Leukemia Tumor Bank, Hôpital Saint Antoine, Paris. The phenotype for HL60 wild-type (CD13^+^, CD15^+^, CD33^+^ CD3^−^, CD14^−^, CD19^−^, HLA-Dr^−^) and for K562 wild-type (BCRAbl^+^ ‘Philadelphia chromosome’, CD13^+^, glycophorin^+^ and CD3^−^, CD19^−^) were determined by flow cytometry. These two cell lines are routinely used as reference cells for chromosome mutation (K562-BCRAbl) and HL60 for leukemia cell endothelial protein-C receptor. All cell lines were cultured using RPMI-1640 containing 10% fetal bovine serum (FBS), 1% L-glutamine 50 U/ml penicillin and 50 μg/ml streptomycin and incubated at 37°C in 5% CO_2_ atmosphere.

### Bone marrow Hospicells

Hospicells were isolated from eight AML bone marrows (Hôpital Hotel Dieu, Paris). The bone marrow-adherent mononuclear cell population (BMMNCs) isolation was carried out by Ficoll density gradient centrifugation as described (21). The contaminating monocytes in the mononuclear cell population were excluded by adhesion on plastic plates for 30 min. Then, the non-adherent cells were collected and cultured for 2 weeks in wells coated with 0.2% gelatin in endothelial cell basal medium MV2 (Promocell, Heidelberg, Germany) supplemented with final concentrations of 1 g/ml ascorbic acid, 10 ng/ml b-FGF, 5 ng/ml EGF, 20 ng/ml insulin growth factor-1 (IGF-1), 0.5 ng/ml VEGF and 15% FBS. The bone marrow Hospicells were isolated as described (2–7).

### Adhesion assay

Adherent BMMNCs (10^5^) were seeded in 6-well plates with their respective culture medium and left to adhere. After completion of cell adhesion, they were washed twice with PBS. HL60 (3×10^6^) were plated onto the adherent cell monolayer at 37°C for 24 min. Then, the supernatant containing non-adherent cells was discarded and the cell monolayer was again washed twice with PBS. The number of BMH in the adherent cell population, obtained after 24 h culture was evaluated. When 5 or more leukemic cells adhered to a single bone marrow stromal cell the latter was considered to be a Hospicell.

### Treatment of cells with IGF-I and IGF-IR inhibitor

The leukemic cell lines were incubated with IGF-I (200 ng/ml) and IGF-IR inhibitor PPP (picropodophyllin, 1 μM) from Calbiochem (Paris).

### Reverse transcription and polymerase chain reaction (RT-PCR)

Total RNA was extracted with the Nucleospin RNA II kit (Macherey-Nagel EURL, Hoerdt, France). Reverse transcription was performed using M-MLV reverse transcriptase and oligo(dt) primers (Gibco-BRL, Paisley, UK). The polymerase chain reaction (PCR) was performed by *Taq* DNA polymerase (Gibco-BRL). Specific primers for *IGF-I* (sense: 5′-AAA TCA GCA G TC TTC CAA C-3′ and antisense: 5′-CTT CTG GGT CTT GGG CAT GT-3′); *IGF-II* (sense: 5′-AGT CGA TGC TGG CTT CTC A-3′ and antisense: 5′-GTG GGC GGG GTCT TGG GTG GGT AG-3′); *IGF-IR* (sense: 5′-GAC ATC CGC AAC GAC TAT CAG-3′ and antisense: 5′-GTA GTT ATT GGA CAC CGC ATC-3′); *IGF-IIR* (sense: 5′-TAC AAC TTC CGG TGG TAC ACC A-3′ and antisense: 5′-CAT GGC ATA CCA GTT TCC TCC A-3′); *MDR1* (sense: 5′-GTT ATA GGA AGT TTG AGT TT-3′ and antisense: 5′-AAA AAC TAT CCC ATA ATA AC-3′); *MRP* (sense: 5′-AAT GCG CCA AGA CTA GGA AG-3′ and antisense: 5′-ACG GGA GGA TGT TGA ACA AG-3′); *MRP2* (sense: 5′-CTG GTT GAT GAA GGC TCT GA-3′ and antisense: 5′-CTG CCA TAA TGT CCA GGT TC-3′); *MRP3* (sense: 5′-GCA GGT GAC ATT TGC TCT GA-3′ and antisense: 5′-CCC TCT GAG CAC TGG AAG TC-3′); *MRP5* (sense: 5′-GGA TAA CTT CTC AGT GGG-3′ and antisense: 5′-GGA ATG GCA ATG CTC TAA AG-3′); *BCRP* (sense: 5′-TTA GGA TTG AAG CCA AAG G-3′ and antisense: 5′-TAG GCA ATT GTG AGG AAA ATA-3′) and *β2-microglubulin* (sense: 5′-CCA GCA GAG AAT GGA AAG TC-3′ and antisense: 5′-GAT GCT GCT TAC ATG TCT CG-3′). The PCR products, along with a 100-bp DNA ladder, were analysed by electrophoresis on agarose gels containing ethidium bromide.

### IGF expression by leukemic cells

The presence of proteins belonging to IGF family in cell lines was revealed by immunocytochemistry. Cells were seeded and fixed at 20,000 cells/well in glass bottom chamber slides (Nunc, Lab-Tek, Naperville, IL, USA). They were then permeabilized and incubated for 2 h at room temperature either with specific primary antibodies (dilution 1/200) anti-IGF-I, -II, -IR or -IIR (R&D Systems, Minneapolis, MN, USA). After several washes, the cells were incubated successively with biotinylated secondary antibody and streptavidine coupled to fluorescein isothiocyanate (dilution 1/500), for 45 min. Isotypic controls were performed concurrently and nuclei were DAPI-labeled. The cells were then visualized by fluorescence microscopy.

### Analysis of P-gp expression

P-gp was studied by using UIC2 (Immunotech, France), monoclonal antibody, followed by labeling with a secondary antibody conjugated with phycoerythrin. Cells (1×10^6^) were fixed and permeabilized using IntraPrep™ (Beckman Coulter, Villepinte, France) as per the manufacturer’s instructions. Fluorescence was measured and analyzed by flow cytometry. Protein expression for each transporter was quantified as the mean fluorescence intensity (MFI) shift (ratio of the MFI of antibody and isotype control). All experiments were performed in triplicate.

### Studies of drug resistance of HL60 and HL60/Dnr cells in the presence of Hospicells

The Hospicells were seeded first at 60% confluency in a 96-well flat-bottomed culture plate with complete medium containing 10% FBS. After 18 h incubation, the leukemic cells (10,000 cells/well) were added and co-cultured for 24 h with Hospicells in the presence of IGF-I (200 μg/ml) or IGF-IR inhibitor (1 μM before addition of daunorubicin). The effect of these cytotoxic agents was evaluated by optical density (OD) measurement using the multi-well plate reader (Wallac, Perkin-Elmer, Waltham, MA, USA). The result is representative of three independent experiments.

### Statistical analyses

The results are presented as mean ± SE and data were analyzed using Student’s t-test P-value (<0.05 was considered significant).

## Results

### Expression of IGFs and IGF-R by leukemic cells

**Figure 1:**
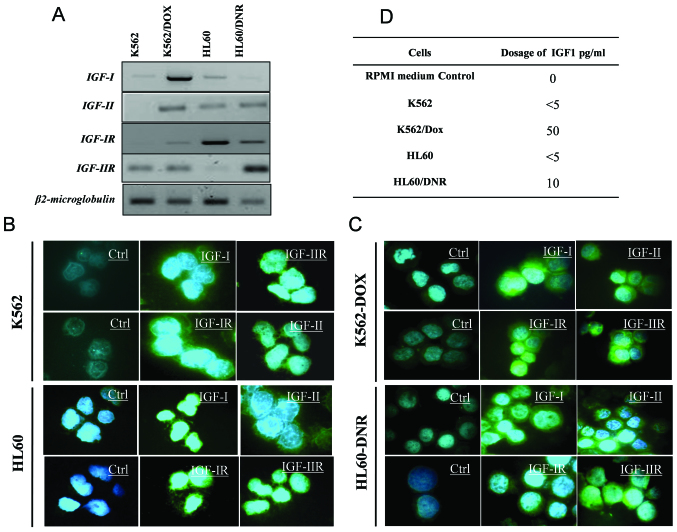
Expression of IGFs and IGF-R by the leukemic cells. *IGF-I*, *-II*, *-IR* and *-IIR* mRNA expression was studied by RT-PCR. *β2*-microglobulin was used as control (A). The proteins of IGF family were analyzed by immunocytochemistry. As negative controls, the cells were incubated only with the secondary antibodies (B). The amount of IGF-I pg/ml in the supernatants of the sensitive and resistant leukemic cells was analyzed by ELISA (C).

Fig. 1A shows the four cell lines (HL60 and K562 sensitive and resistant) the difference in transcription levels of IGFs and their receptor genes. These results were confirmed at the protein level by immunocytochemistry (Fig. 1B and C). Fig. 1D indicates the amount of IGF-I in the supernatants of the sensitive and resistant cells. The results show that the resistant cells secrete more IGF-I (50 pg/ml) than the sensitive cells (<5 pg/ml).

### Expression of P-gp by the sensitive and resistant cells

**Figure 2:**
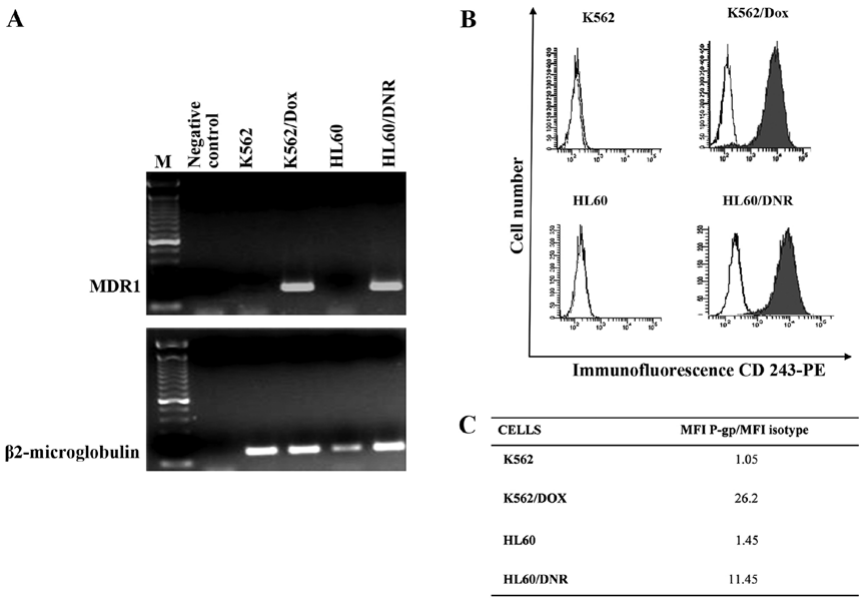
Expression of P-gp by the leukemic cells. MDR1 mRNA expression was studied in K562, K562/Dox, HL60 and HL60/Dnr cells by RT-PCR using *β2*-microglobulin as control (A). Flow cytometry analysis of P-gp in these cells using anti-CD243-phycoerythrin (B). P-gp expression was quantified as the mean fluorescence intensity (MFI) shift (ratio of the MFI of P-gp and isotype control) (C).

The *MDR1* gene coding P-gp protein was studied by the RT-PCR. Fig. 2A shows that MDR1 mRNA is strongly expressed by resistant cells whereas the sensitive cells not at all. These results were confirmed by flow cytometry of P-gp protein (Fig. 2B). The ratios of the Mean Fluorescence Intensity (MFI) of P-gp in resistant cells as against the value for controls are presented in Fig. 2C. Thus it can be inferred that the drug expulsion mechanism is active in the resistance cells and not in the controls.

### The effect of IGF-I on cell proliferation

**Figure 3:**
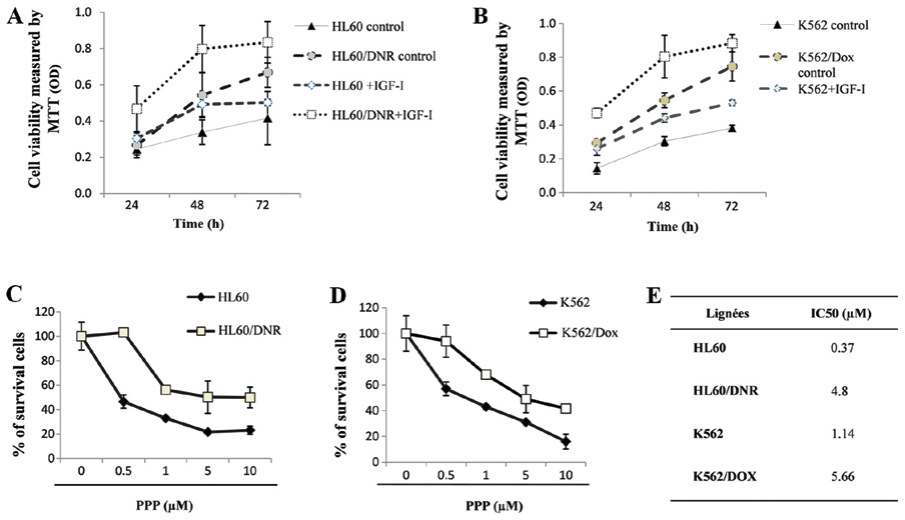
Effect of IGF-I on the proliferation of sensitive and resistant cells. K562 (A) and HL60 (B) were incubated in a culture medium without FBS (control) or in the presence of IGF-I (200 ng/ml) during 24, 48 and 72 h at 37°C. Cell proliferation was studied by MTT. Effects of the inhibitor of IGF-IR (PPP) at various concentrations (0, 0.5, 1, 5 and 10 μM) on the proliferation of sensitive and resistant K562 (C) and HL60 leukemic cells (D). The values of the IC_50_ of PPP μM is presented for each cell type (E).

The effect of IGF-I on the proliferation rate of sensitive K562 and HL60 and resistant K562/Dox and HL60/Dnr cells are shown (Fig. 3A and B). The rate of proliferation of the sensitive cells was lower than that of the resistant ones. These results show that IGF-I promotes proliferation, its effect being more important in the resistant cells than in the sensitive cells.

We also determined the effect of the cyclolignan PPP, an IGF-IR inhibitor (22), on the resistant cells which express P-gp: HL60/Dnr (Fig. 3C) and K562/Dox (Fig. 3D) and sensitive cells K562 and HL60 which do not. The values of the IC_50_ of PPP in these cells were evaluated by MTT tests. Fig. 3E (see table inserted) shows that the value of the IC_50_ of PPP is higher in the resistant cells as compared to sensitive cells.

### IGF-I mediated expression of MDR1 mRNA and P-gp in the sensitive and resistant cells

**Figure 4:**
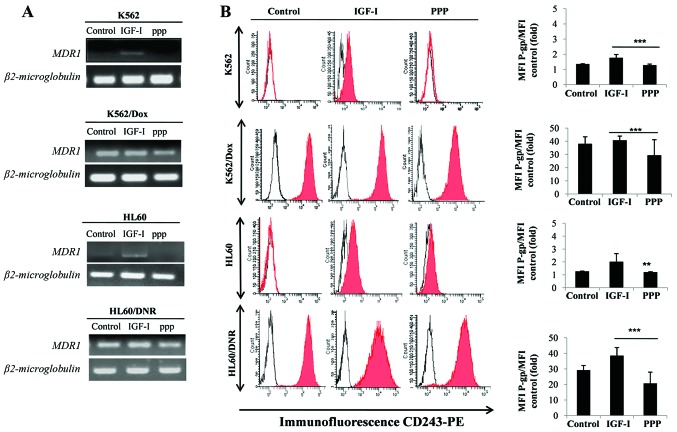
IGF-I controls the expression of *MDR1* and P-gp in leukemic cells. The cell lines were incubated in the presence of IGF-I or PPP for 12 h. The expression of *MDR1* was analyzed by RT-PCR (A). The expression of P-gp protein was studied by flow cytometry using anti-CD243-PE. Isotypic IgG2a was used as negative control (B).

Fig. 4A shows induction of MDR1 mRNA expression and its protein P-gp (Fig. 4B) in cells treated by IGF-1. This expression of P-gp, induced by IGF-I, is inhibited by the presence of PPP in both resistant and sensitive cells. The results suggest that IGF-I, via IGF-IR, is able to induce and control the expression of P-gp, the major protein implicated in chemoresistance.

### Regulation of ABC genes by IGF-I

**Figure 5:**
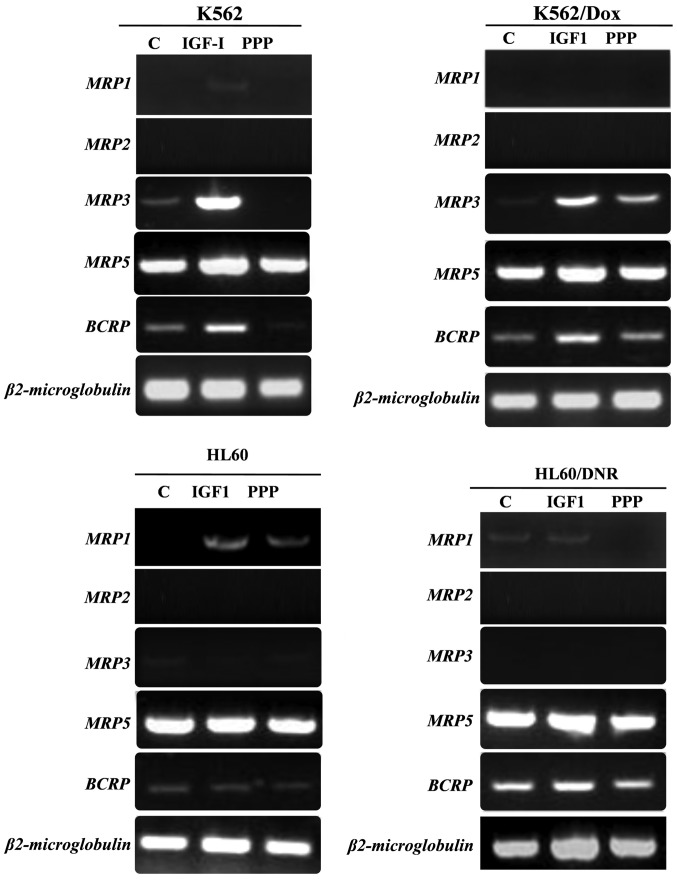
Regulation of the ABC genes by IGF-I. The four leukemic cell lines were incubated in the presence of IGF-I (200 ng/ml) or PPP (0.5 μM for the HL60 and K562; 1 μM for K562/Dox and HL60/Dnr) for 12 h at 37°C. Then, a RT-PCR was carried out to study the expression of mRNAs for *MRP1*, *MRP2*, *MRP3*, *MRP5* and *BCRP* with *β2*-microglobulin as a control.

Normally, the expression of *MRP1*, *MRP2*, *MRP3* and *BCRP* is low or non-existent in the 4 cell lines (K562, K562/Dox, HL60 and HL60/Dnr), whereas *MRP5* is strongly expressed (Fig. 5). In the K562 and its derivative K562/Dox cells, the expression of *MRP3* and *BCRP* increased significantly in the presence of IGF-I. On the other hand, the expression of MRP3 and BCRP, in these same cells decreases or disappears in the presence of PPP.

In HL60/Dnr, we found no significant difference in the expression of MRP1 mRNA in the presence of IGF-I, however, exposure to PPP in the medium led to the inhibition of *MRP1* gene expression. We found no difference in the expression of BCRP and MRP2 mRNAs in the HL60 cells and its derivative HL60/Dnr. Whereas, the expression of *MRP5* gene was not affected by addition of IGF-I nor that of PPP.

### Expression of IGF, IGF-R and MDR1 by Hospicells

**Figure 6:**
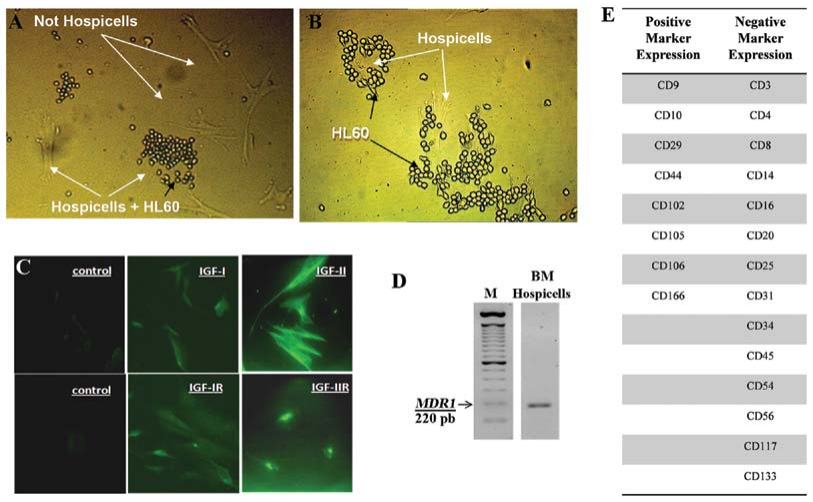
Expression of IGFs, IGF-R and *MDR1* in Hospicells from AML bone marrow. Hospicells were identified among other stromal cells because of their property to interact with HL60 cells (A). Interaction of HL60 with enriched Hospicells (B). The Hospicells were stained by IGF-I, -II, -IR and -IIR antibodies (C). Expression of *MDR1* by Hospicells was analyzed by RT-PCR (D).

Hospicells from the stroma were identified because of their property to interact with HL60 cells. Fig. 6A shows HL60 interaction with a subset of stromal cells (Hospicells) and Fig. 6B with the enriched population of Hospicells. These cells express IGF-I, IGF-II, IGF-IR and IGF-IIR, as studied by immunocytochemistry (Fig. 6C) and P-gp (MDR-1) mRNA by RT-PCR (Fig. 6D).

### Transcriptional regulation of the ABC genes through the association of HL60 with Hospicells

**Figure 7:**
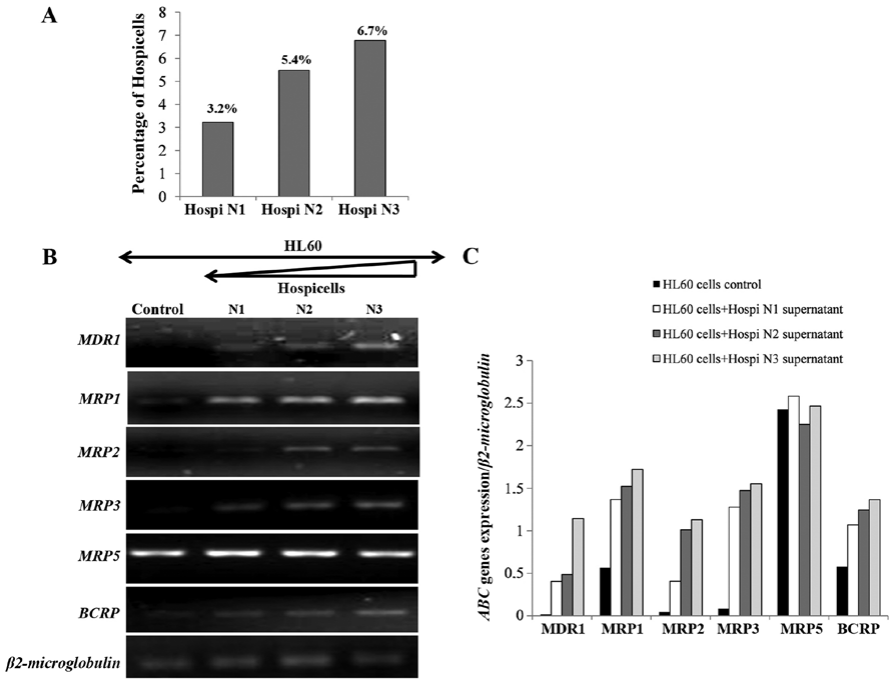

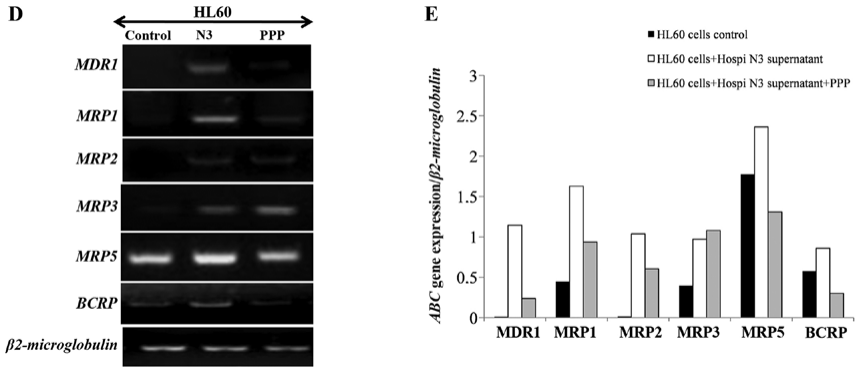
Expression of the ABC genes by HL60 cells co-cultivated with Hospicells. The Hospicells from three bone marrows of AML were counted and expressed as a percentage compared to the total number of mesenchymal stromal cells (A). HL60 cells co-cultivated with the Hospicells of three bone marrows for 12 h and the expression of ABC genes were analyzed by RT-PCR (B). The ratio of ABC gene expression/*β2*-microglobulin is presented in (C). HL60 cells co-cultivated with Hospicells from AML (N3) bone marrow for 12 h in presence or not of PPP, an RT-PCR was carried out to study the ABC gene expression: *MDR1*, *MRP1*, *MRP2*, *MRP3*, *MRP5* and *BCRP* (D). The ratio of ABC gene expression/*β2*-microglobulin is presented in (E).

The number of Hospicells was counted in each bone marrow stroma preparation. It varied from 3 to 9.7% of the total stromal cells. In normal bone marrow only 0.7% of stromal cells are Hospicells (data not shown). Three samples were chosen from AML bone marrow depending on the number of Hospicells (Fig. 7A). Fig. 7B shows that HL60 cells express mRNA for *MDR1*, *MRP2* and *MRP3* in the presence of the Hospicells whereas it was not expressed in control cells. Moreover, the level of expression of ABC genes was directly related to the number of Hospicells present initially in the three BM samples. However, no difference in the expression of *MRP5* in the presence of the Hospicells compared to the control was seen (Fig. 7C).

Fig. 7D and E show that HL60 cells co-cultivated with Hospicells from AML (N3 rich in Hospicells), express the ABC genes. On the other hand, the addition of PPP decreased the expression of *MDR1* and *MRP1* as also the *BCRP*.

These results are interesting since it may suggest that IGF-I was secreted by Hospicells or HL60 cells and acts through autocrine and/or paracrine mechanisms on *MDR1*, *MRP1*, *MRP2* and *BCRP* genes.

### Interaction between HL60 and Hospicells in the presence of IGF-I and induction of chemoresistance

**Figure 8:**
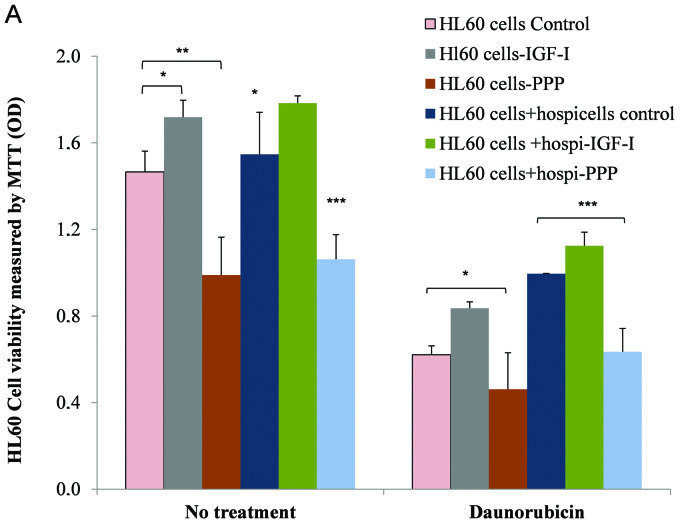

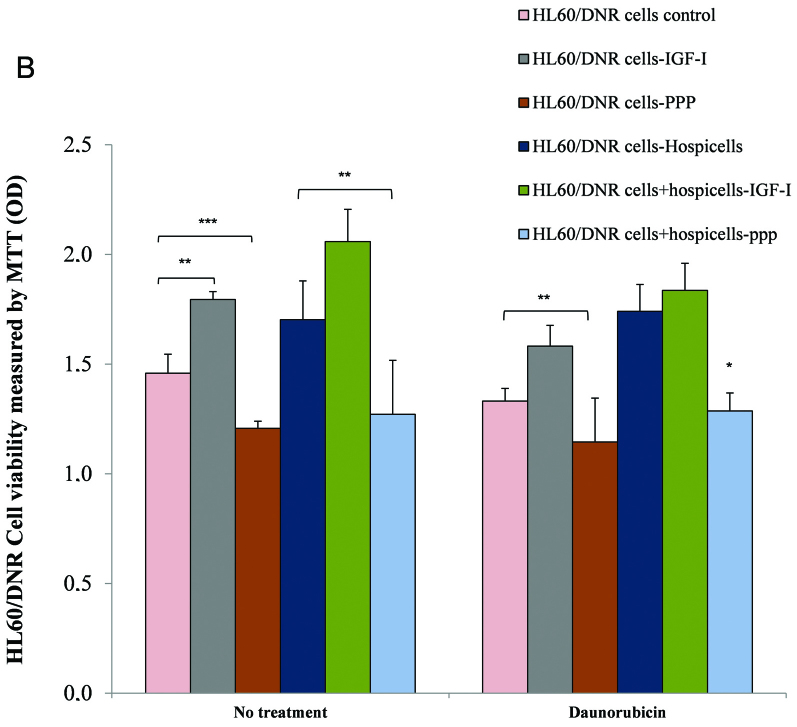
Hospicells protect HL60 cells against daunorubicin effect in the presence of IGF-I. HL60 was co-cultivated with Hospicells in the presence of IGF-I alone or with PPP. The chemotherapy agent, daunorubicin was added to the culture medium for 24 h (A). HL60/Dnr cells co-cultivated with Hospicells from AML N3 in the presence of IGF-I or PPP during 24 h and were incubated thereafter with daunorubicin (B). Average ± SE, (n=3), the difference in expression is significant (^*^P<0.05, ^***^P<0.0005).

Our results show that under culture conditions (without treatment), HL60 sensitive cell proliferation is stimulated by IGF-I (P=0.02) whereas this strongly diminishes in the presence of PPP (P=0.002). This indeed confirms our earlier results (Fig. 8A). In the presence of Hospicells, we observed a significant increased proliferative activity of HL60 cells (P=0.03) as compared to controls. HL60 cells when in physical contact with the Hospicells display a clear resistance to daunorubicin. This resistance is reinforced further by IGF-I (P=0.03) whereas it decreases in the presence of PPP (P=0.02).

These observations confirm that, on one hand IGF-I regulates ABC gene expression (mainly *MDR1*), whereas Hospicells provide protection to HL60 leukemic cells against the effect of chemotherapy. Fig. 8B depicts the interaction between HL60/Dnr cells and Hospicells in the presence of IGF-I and the enhancement of chemoresistance. It shows that proliferation of these resistant cells is stimulated by IGF-I (P=0.0002), whereas it decreases in the presence of PPP (P=0.0003). Thus the interaction between Hospicells and HL60/Dnr cells increases the proliferation of the resistance cells which is further amplified by the presence of IGF-I. The inhibition of IGF-IR by the addition of PPP reduces this drug resistance.

## Discussion

Of the two insulin-like growth factors, IGF-I and IGF-II, IGF-I binds to two distinct cell surface receptors named IGF receptor-I (IGF-IR) and the insulin receptor (INSR). The binding of IGF-I/IGF-IR occurs at a higher affinity than IGFI/INSR binding. IGF-IR is a transmembrane tyrosine kinase receptor and is activated by IGF-I and IGF-II (24–27).

IGF-II binds to the IGF-II receptor and acts as a signaling antagonist. IGF-I and IGF-II form complexes with insulin-like growth factor binding protein (IGFBP). Integrin αvβ3 also plays an important role in IGF-IR signaling and its biological functions (28). Several studies have shown that IGF-I is involved in the progression of malignant hematologic diseases (23).

During this study, the characteristics of the myeloid cell lines, both sensitive and resistant to the agents of chemotherapy, were defined by studying the expression of *MDR1* and its protein P-gp. In addition, the proliferative effect of IGF-I on sensitive or resistant cells and the effect of the IGF-IR inhibitor (PPP) were ascertained. It was noted that the basal level of proliferation of the resistant cells was well above that of the sensitive cells. Exogenous addition of IGF-I stimulated proliferation of the two cell lines without marked difference.

The degree of resistance displayed by HL60 (in contact with Hospicells) was more pronounced when the proportion of Hospicells in givens stroma was high. This was partially abolished by the addition of PPP. However, MRP5 was an exception and remained unchanged. The addition of IGF-I increased the expression of P-gP which could be inhibited again by PPP. The co-cultures of Hospicells with sensitive leukemic cells showed that the Hospicells were able to transmit the character of drug resistance to the sensitive cells. We had previously provided evidence that bone marrow stromal cells secrete significant amounts of FGF (fibroblast growth factor), SDF-1α (stromal cell-derived factor 1) and IGF-1 (8). In the present study we report that bone marrow Hospicells also secrete IGF-1. Consequently, IGF (via autocrine or paracrine pathway) could be considered a strong candidate for the regulation of ABC genes and chemoresistance of HL60 cells.

In an unpublished study we had observed that the interaction of cancer cells with BMH was integrin-dependent. Cell-cell interaction activates integrin signaling pathway which play a crucial role in IGF signaling (28). The IGF-IR and its ligands may also promote growth of metastatic cells in the bone, the preferred site of metastases (29). Recently, we reported that Hospicells from solid tumors can be involved in chemoresistance via oncologic trogocytosis, i.e., through transfer of MDR proteins onto the incoming cancer cells (2) or upregulation of the ABC genes (3). The IGFs are among the more abundant growth factors in bone tissue and are synthesized by various bone marrow cells including stromal cells (8) and BMH. Within the microenvironment, the stromal Hospicells, in concert with IGF-1, provide strong synergetic effect for the maintenance and proliferation of cancer cells. In addition, BMH may represent a niche for homing of cancer cells and the secretion of IGF-1 which provide protection while promoting their proliferation and chemoresistance.

Our results demonstrate that tumor stromal cells contribute in the physiopathological response to IGF-1/IGF-1R. Targeting IGF-1R in multiple myeloma (30) and breast cancer (31) indicated a decrease in the formation of tumors and a diminuation of angiogenesis. In view of the scant attention given so far to the role of microenvironment, in the behavior and biology of the leukemic cells, we have focused attention in the present study on the intimate relationships between Hospicells, IGF-1 and malignant cells.

IGF and IGF1R expression levels are relevant indicators of tumor stage and/or disease progression. Additional studies will be necessary to further clarify the mode of action of the ABC pumps as also other pumps such as LRP (lung resistance-related protein). Curiously IGF seems not to have any effect on MRP5 gene expression.

In conclusion our results suggest the importance of the microenvironment and the IGF-I pathway in drug resistance of leukemic cells. A better knowledge of this close relationship can be helpful in the search for new openings in cancer therapy.
